# Male gender is a predictor of higher mortality in hospitalized adults with COVID-19

**DOI:** 10.1371/journal.pone.0254066

**Published:** 2021-07-09

**Authors:** Ninh T. Nguyen, Justine Chinn, Morgan De Ferrante, Katharine A. Kirby, Samuel F. Hohmann, Alpesh Amin

**Affiliations:** 1 From the Department of Surgery, University of California, Irvine Medical Center, Orange, California, United States of America; 2 From Edwards Lifesciences, Irvine, California, United States of America; 3 From the Department of Statistics, University of California, Irvine Medical Center, Orange, California, United States of America; 4 From Vizient, Centers for Advanced Analytics and Informatics and Department of Health Systems Management Rush University, Chicago, Illinois, United States of America; 5 From the Department of Medicine, University of California, Irvine Medical Center, Orange, California, United States of America; Erasmus Medical Centre: Erasmus MC, NETHERLANDS

## Abstract

**Introduction:**

The coronavirus disease 2019 (COVID-19) pandemic continues to be a global threat, with tremendous resources invested into identifying risk factors for severe COVID-19 illness. The objective of this study was to analyze the characteristics and outcomes of male compared to female adults with COVID-19 who required hospitalization within US academic centers.

**Methods:**

Using the Vizient clinical database, discharge records of adults with a diagnosis of COVID-19 between March 1, 2020 and November 30, 2020 were reviewed. Outcome measures included demographics, characteristics, length of hospital stay, rate of respiratory intubation and mechanical ventilation, and rate of in-hospital mortality of male vs female according to age, race/ethnicity, and presence of preexisting comorbidities.

**Results:**

Among adults with COVID-19, 161,206 were male while 146,804 were female. Adult males with COVID-19 were more likely to have hypertension (62.1% vs 59.6%, p <0.001%), diabetes (39.2% vs 36.0%, p <0.001%), renal failure (22.3% vs 18.1%, p <0.001%), congestive heart failure (15.3% vs 14.6%, p <0.001%), and liver disease (5.9% vs 4.5%, p <0.001%). Adult females with COVID-19 were more likely to be obese (32.3% vs 25.7%, p<0.001) and have chronic pulmonary disease (23.7% vs 18.1%, p <0.001). Gender was significantly different among races (p<0.001), and there was a lower proportion of males versus females in African American patients with COVID-19. Comparison in outcomes of male vs. female adults with COVID-19 is depicted in Table 2. Compared to females, males with COVID-19 had a higher rate of in-hospital mortality (13.8% vs 10.2%, respectively, p <0.001); a higher rate of respiratory intubation (21.4% vs 14.6%, p <0.001); and a longer length of hospital stay (9.5 ± 12.5 days vs. 7.8 ± 9.8 days, p<0.001). In-hospital mortality analyzed according to age groups, race/ethnicity, payers, and presence of preexisting comorbidities consistently showed higher death rate among males compared to females (Table 2). Adult males with COVID-19 were associated with higher odds of mortality compared to their female counterparts across all age groups, with the effect being most pronounced in the 18–30 age group (OR, 3.02 [95% CI, 2.41–3.78]).

**Conclusion:**

This large analysis of 308,010 COVID-19 adults hospitalized at US academic centers showed that males have a higher rate of respiratory intubation and longer length of hospital stay compared to females and have a higher death rate even when compared across age groups, race/ethnicity, payers, and comorbidity.

## Introduction

The coronavirus disease 2019 (COVID-19) pandemic continues to spread globally with more than 25 million confirmed cases and nearly 450,000 deaths in the United States (US) [1]. A top priority of the Centers for Disease Control and Prevention (CDC) is to identify risk factors for severe COVID-19 illness that can lead to hospitalization or death [1]. Some of the current known risk factors for hospitalization and death include older age and patients with certain underlying medical conditions such as diabetes, obesity, immunocompromised state, and pulmonary, cardiac, or renal dysfunction [1]. Additionally, several COVID-19 studies have demonstrated that male is a predictor for higher death rate [2–5]. The objective of this study was to analyze the characteristics and outcomes of male compared to female adults with COVID-19 who required hospitalization within US academic centers.

## Materials and methods

The data for this study were obtained from the Vizient clinical database (CDB/RM^TM^) which is an administrative, clinical, and financial database of more than 650 academic centers and their affiliates. Approval for the use of the data was obtained from Vizient and from the Institutional Review Board of the University of California, Irvine as exempted status.

Discharge records of male and female adults (≥18 years) with a diagnosis of COVID-19 between March 1, 2020 and November 30, 2020 were reviewed. COVID-19 diagnosis was identified using International Classification of Disease, Tenth edition code of U07.1. Outcome measures included demographics, characteristics, length of hospital stay, rate of respiratory intubation and mechanical ventilation, and rate of in-hospital mortality of male vs female according to age, race/ethnicity, and presence of preexisting comorbidities. Race/ethnicity was self-reported by the patient. Chi-Square Test of Independence was performed to determine if statistically significant associations exist between categorical variables. No post hoc analyses were performed. P-values were not adjusted for multiple comparisons. Statistical significance was set at P <0.05. R version 4.0.3 was used for statistical analysis.

## Results

Among adults with COVID-19 discharged during this time period, 161,206 (52.3%) were male while 146,804 (47.7%) were female. All adult patients admitted and discharged during the time period with a diagnosis of COVID-19 as described in the Methods were included in our analysis. Differences in characteristics and demographics of male vs. female adults with COVID-19 are depicted in Table 1. Adult males with COVID-19 were more likely to have hypertension (62.1% vs 59.6%, p <0.001%), diabetes (39.2% vs 36.0%, p <0.001%), renal failure (22.3% vs 18.1%, p <0.001%), congestive heart failure (15.3% vs 14.6%, p <0.001%), and liver disease (5.9% vs 4.5%, p <0.001%). Adult females with COVID-19 were more likely to be obese (32.3% vs 25.7%, p<0.001) and have chronic pulmonary disease (23.7% vs 18.1%, p <0.001). Gender was significantly different among races (p<0.001), and there was a lower proportion of males versus females in African American patients with COVID-19. Comparison in outcomes of male vs. female adults with COVID-19 is depicted in Table 2. Compared to females, males with COVID-19 had a higher rate of in-hospital mortality (13.8% vs 10.2%, respectively, p <0.001); a higher rate of respiratory intubation (21.4% vs 14.6%, p <0.001); and a longer length of hospital stay (9.5 ± 12.5 days vs. 7.8 ± 9.8 days, p<0.001). In-hospital mortality analyzed according to age groups, race/ethnicity, payers, and presence of preexisting comorbidities consistently showed higher death rate among males compared to females (Table 2). Adult males with COVID-19 were associated with higher odds of mortality compared to their female counterparts across all age groups, with the effect being most pronounced in the 18–30 age group (OR, 3.02 [95% CI, 2.41–3.78]).

**Table 1:** Summary of demographics and characteristics of male vs female adults with COVID-19.

Demographics and Characteristics	Male (n = 161,206)	Female (n = 146,804)	P Value*
Age group, No. (%)			<0.001
18–30	7795 (4.8)	14837 (10.1)	
31–50	34184 (21.2)	29091 (19.8)	
51–64	47429 (29.4)	34559 (23.5)	
65–74	34820 (21.6)	27695 (18.9)	
75–84	24871 (15.4)	23565 (16.1)	
≥ 85	12107 (7.5)	17057 (11.6)	
Race/Ethnicity, No. (%)			<0.001
Caucasian	82385 (51.1)	72983 (49.7)	
African American	35616 (22.1)	38656 (26.3)	
Asian	5202 (3.2)	4646 (3.2)	
Hispanic*	44127 (27.4)	37749 (25.7)	
Other	38003 (23.6)	30519 (20.8)	
Payer No. (%)			<0.001
Commercial	42150 (26.1)	34371 (23.4)	
Medicare/Medicaid/State-assisted	102833 (63.8)	102619 (69.9)	
Existing comorbidities, No. (%)			
Obesity	41406 (25.7)	47452 (32.3)	<0.001
Hypertension	100,163 (62.1)	87546 (59.6)	<0.001
Diabetes	63,209 (39.2)	52900 (36.0)	<0.001
Anemia	32497 (20.2)	31898 (21.7)	<0.001
Chronic pulmonary disease	29120 (18.1)	34821 (23.7)	<0.001
Renal failure	35993 (22.3)	26644 (18.1)	<0.001
Congestive heart failure	24601 (15.3)	21434 (14.6)	<0.001
Liver disease	9499 (5.9)	6600 (4.5)	<0.001

* comparing male vs female, Chi-square tests

**Table 2:** Outcomes of male vs female adults with COVID-19.

Outcomes	Male (n = 161,206)	Female (n = 146,804)	P Value*	OR (Male vs female)	95% CI
Overall in-hospital mortality, No. (%)	22,266 (13.8)	14940 (10.2)	<0.01		
In-hospital mortality according to age group, No. (%)					
18–30	198 of 7795 (2.5)	127 of 14837 (0.9)	<0.01	3.02	2.41, 3.78
31–50	1739 of 34184 (5.2)	803 of 29091 (2.8)	<0.01	1.89	1.73, 2.06
51–64	4935 of 47429 (10.4)	2561 of 34559 (7.4)	<0.01	1.45	1.38, 1.53
65–74	5989 of 34820 (17.2)	3747 of 27695 (13.5)	<0.01	1.33	1.27, 1.39
75–84	5849 of 24871 (23.5)	4071 of 23565 (17.3)	<0.01	1.47	1.41, 1.54
≥ 85	3556 of 12107 (29.4)	3631 of 17057 (21.3)	<0.01	1.54	1.46, 1.62
In-hospital mortality according to race/ethnicity, No. (%)					
Caucasian	11802 of 82385 (14.3)	7730 of 72983 (10.6)	<0.01		
African American	4465 of 35616 (12.5)	3803 of 38656 (9.8)	<0.01		
Asian	809 of 5202 (15.6)	527 of 4646 (11.3)	<0.01		
Hispanic*	5668 of 44127 (12.8)	3103 of 37749 (8.2)	<0.01		
Other	5190 of 38003 (13.7)	2880 of 30519 (9.4)	<0.01		
In-hospital mortality according to payers, No. (%)					
Commercial	3476 of 42150 (8.2)	1572 of 34371 (4.6)	<0.01		
Medicare/Medicaid/State-assisted	17296 of 102833 (16.8)	12830 of 102619 (12.5)	<0.01		
In-hospital mortality according to preexisting comorbidities, No. (%)					
Obesity	5773 of 41406 (13.9)	4836 of 47452 (10.2)	<0.01		
Hypertension	16848 of 100,163 (16.8)	11751 of 87546 (13.4)	<0.01		
Diabetes	10642 of 63,209 (16.8)	7209 of 52900 (13.6)	<0.01		
Anemia	6442 of 32497 (19.8)	4700 of 31898 (14.7)	<0.01		
Chronic pulmonary disease	4988 of 29120 (17.1)	4401 of 34821 (12.6)	<0.01		
Renal failure	8081 of 35993 (22.5)	5206 of 26644(19.5)	<0.01		
Congestive heart failure	5722 of 24601 (23.3)	4391 of 21434 (20.5)	<0.01		
Liver disease	1484 of 9499 (15.6)	848 of 6600 (12.8)	<0.01		
Rate of respiratory intubation & mechanical ventilation (%)	34452 (21.4)	21414 (14.6)	<0.01		
Mean length of hospital stay (days)	9.5 ± 12.5	7.8 ± 9.8	<0.01		

## Discussion

This large analysis of 308,010 adults with COVID-19 hospitalized at US academic centers showed that males have higher death rate compared to females. This finding was consistently observed across all age groups, race/ethnicity, payers, and preexisting comorbidities. Our finding is in concordance with other studies showing that male sex is a strong predictor for higher risk of death in hospitalized adults with COVID-19 [2–5]. The CDC has reported that 54% of COVID-19 deaths have been among men [1]. In a meta-analysis of 3,111,714 reported global cases, male patients had a higher odds of death compared to female patients (Odds ratio = 1.4; 95% confidence interval = 1.31, 1.47) [5]. This raises the question of the importance of co-morbidities as drivers for mortality risk leading to the differences noted between sexes or differences in the immune system response between sexes [3]. Surprisingly, although the presence of pulmonary disease was higher in our female cohort, the mortality remained higher in males even when accounting for this comorbidity. The association of higher odds of mortality in males with COVID-19 compared to female across age groups also raises the question of the role of sex hormones in immune response, and the effect that age has on sex hormone concentration [6]. There are some limitations to this retrospective study. Data for this study is based on the Vizient database which has potential for misclassification and inaccuracy of coding, and missing data. The designation of male vs. female sex in the Vizient database is self-reported which has the potential for subjective bias. Despite these limitations, this study provides data from a large cohort of adults with COVID-19 documenting the consistent association of males with higher death rate. Further studies are needed to determine the underlying mechanisms for this differential risk of death between male vs. female adults with COVID-19.

## Supporting information

S1 File (XLSX)

S2 File (XLSX)

S3 File (XLSX)

S4 File (XLSX)

S5 File (XLSX)

S6 File (XLSX)

S7 File (XLSX)

S8 File (XLSX)

S9 File (XLSX)

S10 File (XLSX)

S11 File (XLSX)

S12 File (XLSX)

S13 File (XLSX)

S14 File (XLSX)
